# Surgical Treatment of Lateral Tibial Plateau Fractures Involving the Posterolateral Column

**DOI:** 10.1111/os.12544

**Published:** 2019-11-06

**Authors:** Qi‐jie Shen, Jin‐li Zhang, Guo‐sheng Xing, Zhong‐yu Liu, En‐qi Li, Bao‐cheng Zhao, Yu‐chen Zheng, Qing Cao, Tao Zhang

**Affiliations:** ^1^ Department of Orthopaedics Tianjin Hospital Tianjin China; ^2^ Tianjin Institute of Orthopedics of Integrated Traditional Chinese and Western Medicine Tianjin Hospital Tianjin China

**Keywords:** Approaches, Posterolateral column, Surgical treatment, Tibial plateau fracture

## Abstract

**Objective:**

To summarize the indications and the clinical effects of a transfibular neck osteotomy approach and a combined anterolateral and posterolateral approach in the treatment of fractures of the lateral tibial plateau involving the posterolateral column.

**Methods:**

Eleven patients with lateral tibial plateau fractures were included in the present study. The fractures were Schatzker type II or lateral platform fractures involving posterolateral column. The anterolateral combined posterolateral approach (lateral + posterolateral locking plate fixation) was applied in 7 patients and 4 patients underwent transfibular neck osteotomy (lateral + posterolateral locking plate fixation + 1/4 tubular plate edge fixation, fibular osteotomy with Kirschner wire tension band fixation, and hollow nail fixation for upper tibiofibular joint). All cases were followed up for 12–24 months, with an average follow‐up of 17.5 ± 5.0 months. At the last followup, the Rasmussen radiological criteria were used to evaluate the effect of fracture reduction and fixation. The knee joint function was evaluated using the knee function evaluation criteria of the Hospital for Special Surgery (HSS). The Lachman test and the pivot‐shift test were used to evaluate the anterior and posterior and rotational stability of the knee joint. The range of knee motion was recorded.

**Results:**

Bone healing was achieved in all patients with fractures treated with a transfibular neck osteotomy approach and a combined anterolateral and posterolateral approach. At the last follow‐up, both the Lachman test and the pivot‐shift test results were negative. All patients had complete knee extension. For the combined anterolateral and posterolateral approach, the knee flexion angle was 110°–130°, with an average of 122.86° ± 7.56°. For the transfibular neck osteotomy approach, the knee flexion angle was 115°–130°, with an average of 120.00° ± 7.07°. For the patients in which the combined anterolateral and posterolateral approach was used, the Rasmussen score was 12–18 points, with an average of 16.00 ± 2.56 points. The results were excellent in 4 cases and good in 3 cases; therefore, 100% of results were excellent or good. For patients in which the transfibular neck osteotomy approach was used, the Rasmussen score was 10–18 points, with an average of 15.25 ± 3.77 points. The results were excellent in 2 cases, good in 1 case, and acceptable in 1 case; therefore, 75% of results were excellent or good. The HSS score for the combined anterolateral and posterolateral approach was 76–98 points, with an average of 88.43 ± 7.55 points. The results were excellent in 5 cases and good in 2 cases; therefore, 100% of results were excellent or good. The HSS score for the transfibular neck osteotomy approach was 74–96 points, with an average of 87.25 ± 9.43 points. The results were excellent in 3 cases and good in 1 case; therefore, 100% of results were excellent or good. There were no significant differences in operation time, surgical blood loss, fracture healing time, postoperative imaging score, and knee function evaluation between the two approaches. One patient who underwent transfibular neck osteotomy had a 3‐mm step that gradually appeared, but no significant abnormalities were found in the width of the platform and the lower limb force line. One patient in whom the combined anterolateral and posterolateral approach was used showed numbness in the common peroneal nerve. No common peroneal nerve injury occurred through the transfibular neck osteotomy approach.

**Conclusions:**

The anterolateral combined posterolateral approach and the transfibular neck osteotomy approach are effective in the surgical treatment of lateral tibial plateau fractures involving the posterolateral column. However, the transfibular neck osteotomy approach is more suitable for the posterolateral plateau articular surface damaged with bone separation and displacement, deep collapse, cases involving a large range of the posterolateral column, especially fractures of the lateral tibial plateau in the upper tibiofibular syndesmosis area of the line connecting the anterior and posterior margin of the fibular head to the midpoint of the plateau.

## Introduction

Lateral tibial plateau fractures are common fracture types in tibial plateau fractures, belonging to the Schatzker type I, II, and III fractures, of which 50.4% involve the posterolateral aspect of the lateral platform^1^. According to the three‐column classification of tibial plateau fractures based on Luo *et al*., the lateral tibial plateau fracture include the lateral column and the posterior column fracture. The posterolateral column fracture is a special type of lateral tibial plateau fracture^2^, ^3^, which is usually caused by the femoral lateral condyle hitting one third of the rear of the lateral platform after being subjected to axial and valgus violence, when the knee was flexed or semi‐flexed^4^. Although currently there are no reports on the extent of posterolateral column fractures involving plateaus requiring surgical treatment. However, for the posterolateral split and collapse fracture of the plateau which was caused by this shear force, if it was not effectively reset in time, it may have a potential impact on the posterolateral stability of the knee joint. Later weight‐bearing will often cause the fracture to re‐shift, resulting in knee flexion unstable and abnormal activity^1^, ^5^, ^6^, ^7^. When the joint surface step reaches 3 mm, the local stress will increase by 75%, and as the step increases, the local stress will further increase, the articular cartilage wear will increase, and then the joint degeneration will be aggravated, thus seriously affecting the knee joint function^5^, ^8^.

Most of the lateral tibial plateau fractures are not difficult to treat. However, the posterolateral column of the tibial plateau was special in local anatomy, of which 61.7% was blocked by the fibular head^9^, and adjacent to the iliac vessels popliteal vessels, the common peroneal nerve and the posterolateral complex. The exposure and fixation of the posterolateral column fracture was a difficult point of the surgery, and the treatment of the posterolateral tibial plateau fracture was very challenging^10^, ^11^. At present, there was no uniform standard for the surgical approach for the posterolateral column fracture of the tibial plateau. It was mainly divided into two categories: non‐osteotomy approach and osteotomy approach. The morphological features of the fracture, the extent of the posterolateral column involved, whether to merge other column fractures on the platform, the condition of soft tissue and the surgeon's experience are factors to be considered. Non‐osteotomy approaches such as anterolateral^3^ or posterolateral^12^, ^13^ are optional surgical approaches. But such surgical approaches have limitations^14^, ^15^, ^16^ with limited exposure and they can only be fixed by a single plate. Thus they have their own indications and most of which are suitable for the posterolateral collapse or split fracture of the simple tibial plateau. For the posterolateral column and the lateral column involving the lateral tibial plateau collapse fracture, the lateral column and the posterolateral column are often required to be restored together, and the anterolateral and posterior double plates are firmly fixed, and the single approach was obviously unable to meet the surgical needs^17^, ^18^. The combine anterolateral and posterolateral approach^18^, ^19^ have the advantages of the anterolateral and posterolateral single incisions. The lateral and posterolateral column fractures are exposed before and after the fibular head, and the reduction was completed and fixed with two plates. Through the fibular neck osteotomy approach^20^, ^21^, the lateral column and the posterolateral column can be exposed in a larger range, and the fracture can be restored and fixed under direct vision. However, there was no relevant research comparison for the case of when fibular osteotomy was needed.

In view of this, since October 2013, we have treated some cases of lateral tibial plateau fractures involving the posterolateral aspect by anterolateral and posterolateral combined approaches or transfibular neck osteotomy. This article retrospectively analyzed this group of data. The purpose is to: (i) explore the choice of the combined anterolateral and posterolateral approach, transfibular neck osteotomy; (ii) analyze the use of internal fixation; and (iii) summarize surgical complications and prevention.

## Materials and Methods

### *Inclusion and Exclusion Criteria*

Inclusion criteria: (i) lateral tibial plateau fracture including posterolateral column and lateral column fracture; (ii) combined anterolateral and posterolateral approaches or transfibular neck osteotomy are used in surgery; (iii) no vascular and nerve damage, no calf compartment syndrome; (iv) Rasmussen radiological criteria are used to evaluate the effect of fracture reduction and fixation, and the knee function score of the hospital for special surgery was used to evaluate surgical effect; and (v) retrospective case analysis.

Exclusion criteria: (i) open fracture; (ii) 2 weeks after the fracture; (iii) previous knee disease, joint function is limited; (iv) multiple fractures of the lower limbs, affecting the subsequent rehabilitation of the knee joint; and (v) follow‐up time <12 months.

### *General Information*

From October 2013 to December 2017, a total of 11 patients with lateral tibial plateau fractures were included in the present study according to the above inclusion and exclusion criteria. The study included 8 male and 3 female patients; patients were aged 19–61 years old, with an average of 40.5 ± 12.5 years. Causes of injury: 2 cases of traffic accidents, 6 cases of bicycle falls, and 3 cases of high‐level fall injuries. Fractures according to Schatzker classification^22^: 11 cases were type II. Luo *et al*.^10^ three‐column classification: all of them were posterolateral column with lateral column fractures. Fracture morphology according to Chen *et al*.^23^ classification of posterior condylar fracture of tibial plateau: all of them were type IV posterolateral condylar splitting and collapse fractures. Among the 11 patients, 1 patient had a rib fracture and 1 patient had a distal radius fracture. Knee joint injury: 2 cases of fibular capitulum fracture, 1 case of anterior cruciate ligament tibial insertion avulsion, 5 cases of anterior cruciate ligament injury, 2 cases of posterior cruciate ligament injury, 1 case of avulsion and rupture of medial collateral ligament of femoral medial condyle, 4 cases of medial collateral ligament injury, 1 case of lateral collateral ligament injury, and 7 cases of lateral meniscus injury.

### *Preoperative Preparation*

All patients underwent X‐ray, 3D CT, and MR examination before surgery to understand the type of fracture, the location of the fracture block and the comminuted displacement, the degree of collapse of the joint surface, and the soft tissue injury. The 3D CT included CT angiography examination to determine the distance of the anterior tibial artery from the bifurcation of the popliteal artery to the posterior articular surface of the platform and whether there was anatomical variation. All cases were treated with detumescence, pain relief, and low molecular weight heparin to prevent deep venous thrombosis of the lower limbs. Time from injury to surgery was 6–14 days, with an average of 9.8 ± 2.9 days.

### *The Surgical Method*

According to the fracture characteristics and the extent of involvement, we determined the surgical approach and internal fixation to use. The anterolateral combined posterolateral approach was performed in 7 cases: posterolateral column split depression fracture, with articular surface collapse and collapse <12 mm; bone separation and displacement were not obvious. The posterior wall splitting range does not involve the upper tibiofibular syndesmosis area. In 4 cases, fibular neck osteotomy was performed: split depression fracture of the posterolateral column, articular surface comminution, fracture block >3 pieces with bone block separation and displacement, and articular surface collapse >12 mm. The range of the posterior wall fracture line was large and involved the upper tibiofibular syndesmosis area.

General anesthesia or continuous epidural anesthesia was used, and a pneumatic tourniquet was used to control intraoperative bleeding. The tourniquet pressure was maintained at 280 mmHg.

#### *Combined Anterolateral and Posterolateral Approach*

**Figure 1:**
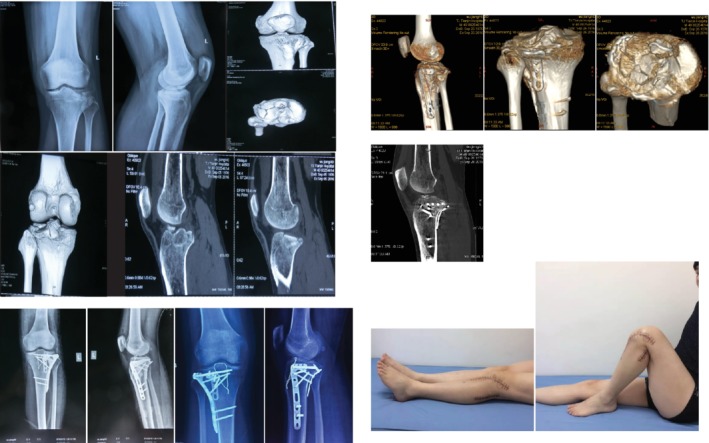
Combined anterolateral and posterolateral approach. Male patient, 40 years old. Schatzker type II fracture of the tibial plateau. The anterolateral column and the posterolateral column of the tibial plateau are involved in this anterior cruciate ligament tibial insertion avulsion fracture. The anterolateral and posterolateral articular surface were reduced and fixed with two locking plates; the tibial avulsion fracture of the anterior cruciate ligament was fixed with wire. The knee joint function was good after surgery.

- Position: The patient was placed in the lateral prone position.
- Incision: The anterolateral incision originates from the posterolateral aspect of the lower part of the patella and was appropriately extended along the tibia to the distal end *via* the Gerdy nodule. The posterolateral incision originates 2 cm above the knee joint line and extends appropriately along the medial posterior margin of the fibula to the distal end.
- Exposure: The anterolateral incision was cut layer by layer, the anterolateral tibial plateau were separated and exposed, the joint capsule was opened, the meniscus was examined, the meniscus was retracted and protected, and the anterolateral fracture block and articular surface were exposed. In the deep posterolateral incision, the lateral head of the gastrocnemius muscle was pulled inward, the inferior lateral genicular artery was ligated, it means enter through the space between two muscles. Care was taken to protect the bifurcation of the popliteal artery to the anterior tibial artery approximately 3 cm below knee surface to reveal the lateral part of the tibial plateau.
- Reduction: The anterolateral and posterolateral collapse of the articular surface bone was restored through the anterolateral fracture line. The Kirschner wire was temporarily fixed, and the fracture reduction was determined under direct vision or by C‐arm fluoroscopy. The posterolateral wall splitting was also reset, and the posterior cortical support was restored.
- Fixation: The anterolateral side was fixed with an “L”‐shaped tibial plateau outside locking steel plate and screw, and the posterolateral side was supported by a “T” shaped steel plate. (A typical case is shown in Fig. 1.)

#### *Transfibular Neck Osteotomy Approach*

**Figure 2:**
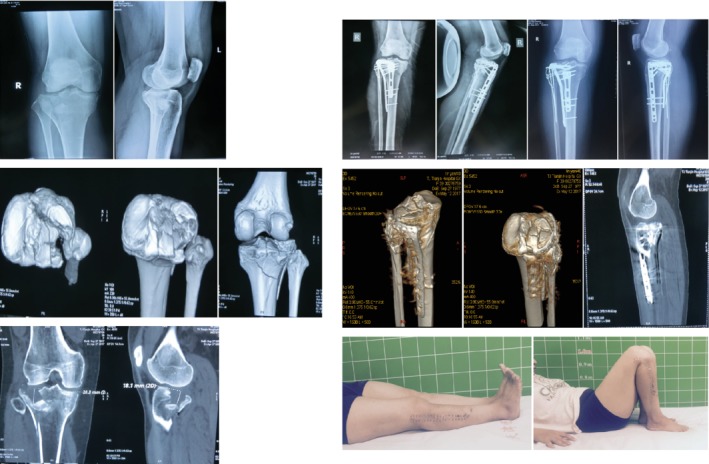
Transfibular neck osteotomy approach. Female patient, 40 years old. Schatzker type II fracture of the tibial plateau. The posterolateral platform fracture collapse and splitting simultaneously exist, the articular surface damage is severely accompanied by bone separation and displacement, and the collapse depth is >12 mm. The anterolateral and posterolateral articular surface were reduced and fixed with two locking plates and 1/4 tube‐shaped plate outer joint edge fixation; the K‐wire tension band was used to fix the fibular osteotomy and the cannulated nail to fix the upper tibiofibular joint. The knee joint function was good after surgery.

- Position: The patient was placed in the lateral decubitus position.
- Incision: The incision was made in a longitudinal line along the fibular head on the outside of the knee joint. The proximal end was 5–8 cm above the plane of the fibular head and extends 10–15 cm distally.
- Revealed: Deep incision of the iliotibial tract was made based on the skin incision, and the common peroneal nerve was revealed on the posterior edge of the biceps femoris. Fully free the common peroneal nerve, and the rubber strips were properly pulled away for protection. The anterior full‐fascial fascia flap was pulled forward, and the anterior tibialis anterior was cut along the anterolateral tibia. The periosteum was peeled off to reveal the anterior lateral surface of the tibia. The proximal end of the fibula was peeled along the fibular muscle to expose the fibular neck at the stop point. The iliotibial tract, the biceps femoris tendon, and the lateral collateral ligament were retained at the fibular head termination, and the common peroneal nerve was protected. The fibula was amputated at the part of the neck of the fibula. Pull the fibula head proximally. The articular capsule and coronary ligament were cut, and the meniscus was retracted proximally to reveal the lateral and posterolateral sides of the tibial plateau.
- Reduction: The fracture and meniscus injury were checked. The collapsed articular surface bone was restored under direct vision, and the Kirschner wire was temporarily fixed. Posterolateral wall fracture was also reset, and the posterior cortical support was restored. Bone defects under the articular surface were filled with allogeneic bone or allogeneic bone combined with artificial bone graft.
- Fixation: The 1/4 tube‐shaped steel plate was attached to the posterolateral joint edge of the tibial plateau after shaping, and the articular surface bone block was fixed by multi‐angle screws, so that the comminuted posterolateral articular surface bone block was fixed as a whole. The anterolateral side was fixed with the “L” shaped tibial plateau outside locking steel plate and screw, and the posterolateral side was supported by the “T” shaped steel plate. During the operation, care should be taken to avoid damage to the attachment points of the lateral collateral ligament and the biceps tendon in the fibular head. The fibular head was turned down and the fibula was fixed with a Kirschner wire tension band to ensure the reduction of the upper tibiofibular joint. The upper tibiofibular joint was fixed by screwing the hollow nail from the fibular head to the proximal tibia (a typical case is shown in Fig. 2).

### *Postoperative Treatment*

A drainage tube was placed in the wound after the operation, and the dressing was pressure‐wrapped after the wound was sutured. Pain relief and anti‐inflammatories were given postoperatively for symptomatic treatment. Low molecular weight heparin was given to prevent deep vein thrombosis 12 h after surgery for 5 weeks. After the anesthesia subsided, patients were encouraged to contract the quadriceps and the ankle flexion and extension. The drainage tube was removed 48 h after the operation, and the Doppler examination of the lower extremities was performed. If it was confirmed that there was no deep vein thrombosis, the patient could perform knee joint activities with the aid of a CPM machine or independently. The range of 0°–90° flexion and extension was reached within 2 weeks. The intensity of functional exercise was gradually increased without weight‐bearing on the affected limb. For patients with ligament injuries, the hinged brace was used for 6 weeks. Cases of numbness in common peroneal nerve innervation areas were treated with nutritional peripheral nerve drugs. Partial weight‐bearing of the affected limb commenced 2–3 months after the operation, and the weight‐bearing time was finally determined according to the degree of fracture comminution and fracture healing.

### *Follow‐up and Efficacy Evaluation Indicators*

The outpatients were reviewed 2, 6, and 12 weeks after surgery, and were followed‐up monthly. The fixation and healing of the fracture were observed by X‐ray and 3D CT examination. Knee flexion was evaluated, as well as extension angle and stability, pain, and related damage recovery.

#### *Rasmussen Radiological Evaluation*

Rasmussen^24^ radiological criteria were used to assess fracture reduction and fixation at the last follow‐up. The assessment includes whether the joint surface has collapsed, whether the ankle has widened, and whether there is a knee or varus deformity; a score of 18 is excellent, 12–17 is good, 6–11 is acceptable, and <6 is poor.

#### *Evaluation of Hospital for Special Surgery Function*

The Hospital for Special Surgery (HSS) knee function evaluation criteria are used for knee joint function assessment^25^. The HSS score reflects pain, functional mobility, muscle strength, knee deformity, stability, and other items, indicating the overall function of the knee joint surgery; a score of 85–100 is excellent, 70–84 is good, 60–69 is acceptable, and below 60 is poor.

#### *Lachman and Pivot‐shift Test*

The anterior and posterior knee rotation and rotational stability were evaluated using the Lachman and Pivot‐shift tests. The range of knee motion was recorded. For the Lachman test, patients were placed in the supine or prone position, with knee flexion 30°; we determined the degree of anterior and posterior movement of the tibia and the end point. By comparison with the healthy side, positive results suggested anterior cruciate ligament or posterior cruciate ligament injury. For the Pivot‐shift test, the patient was placed in a supine position, the knee joint was straightened, the ankle joint was rotated, and the knee joint was everted. When the knee was bent 30°–40° and the tibia was suddenly reset, the result of text was positive was positive.

### *Statistical Analysis*

Data was processed using IBM SPSS 16.0 statistical software. Using the Shapiro–Wilk test, whether the measurement data was normally distributed was assessed, as well as the operation time, the amount of bleeding, and the healing time, the Rasmussen radiological standard evaluation, the HSS knee function evaluation, the knee flexion angle, etc. were normal distribution data, and the homogeneity of variance was expressed as x ± s. Comparison of the two groups of measurement data was performed by two independent samples *t*‐test, and the difference was statistically significant at *P* < 0.05.

## Result

The short‐term (within 2 years) combined anterolateral and posterolateral approach group was close to the transfibular neck osteotomy approach group in regard to operation time, surgical blood loss, fracture healing time, Rasmussen score, HSS score, and knee flexion angle, and the differences were not significantly different (*P* > 0.05). (Table 1).

**Table 1:** Comparison of postoperative outcomes between the two surgical approaches (x ± s)

Surgical approach	Number	Operation time (min)	Surgical blood loss (mL)	Healing time (week)	Rasmuaaen score	HSS score	Knee flexion angle (^o^)
Combined anterolateral and posterolateral	7	114.29 ± 19.88	271.43 ± 56.69	12.43 ± 2.2	16.00 ± 2.56	88.43 ± 7.55	122.86 ± 7.56
Transfibular neck osteotomy	4	157.50 ± 33.04	325.00 ± 50.00	13.50 ± 1.73	15.25 ± 3.77	87.25 ± 9.43	120.00 ± 7.07
*P*‐value	—	0.499	0.566	0.340	0.702	0.824	0.649

HSS, Hospital for Special Surgery.

### *The Operation*

#### *General Situation*

*Combined anterolateral and posterolateral approach*

The operation time was 100–150 min, with an average of (114.29 ± 19.88) min. Intraoperative bleeding was 200–350 mL, with an average of (271.43 ± 56.69) mL.

*Transfibular neck osteotomy approach*

The operation time was 120–200 min, with an average of (157.50 ± 33.04) min. Intraoperative bleeding was 300–400 mL, with an average of (325.00 ± 50.00) mL.

#### *The Treatment of Knee Joint Injury*

Four cases of lateral meniscus tears were sutured; 1 case of bucket handle could not be repaired and removed, and 2 cases showed contusion, but were not treated. One case of tibial avulsion fracture of the anterior cruciate ligament was treated with wire fixation after reduction, and 1 case of avulsion and rupture of the medial collateral ligament in the femoral condyle was treated with astral plate fixation. Four cases of medial collateral ligament injury, 1 case of lateral ligament injury, 5 cases of anterior cruciate ligament injury, and 2 cases of posterior cruciate ligament injury were ligament body I°–II° injuries, and the conservative treatment effect was satisfactory.

### *Fracture Healing*

All 11 patients in this group were followed up for 12–24 months, with an average of 17.5 ± 5.0 months. Bone healing was achieved in all patients. Fracture healing time for the combined anterolateral and posterolateral approach was 10–15 weeks, with an average of 12.43 ± 2.2 weeks; fracture healing time for the transfibular neck osteotomy approach was 12–15 weeks, with an average of 13.50 ± 1.73 weeks. The osteotomy of the fibular neck osteotomy was healed.

### *Bone Grafting and Internal Fixation*

In this group of 11 patients, the bone defect below the articular surface after the reduction of the lateral tibial plateau collapse area was filled with injectable calcium sulfate MIIGTMX3 (Wright, USA) bone material.

For the combined anterolateral and posterolateral approach, 7 cases of the anterolateral side were fixed with the 3.5‐mm system “L” shaped tibial plateau outside locking steel plate and screw (Synthes, Switzerland), and the posterolateral side was supported by the 3.5‐mm system “T” shaped steel plate (Synthes, Switzerland).

For the transfibular neck osteotomy, 4 cases of the anterior lateral side were fixed with the 3.5‐mm system “L” shaped tibial plateau outside locking steel plate and screw (Synthes, Switzerland); 2 cases of the posterolateral side were supported by the 3.5‐mm system “T” shaped steel plate (Synthes, Switzerland), and two cases were supported by the 2.7‐mm system 1/4 tube‐shaped steel plate (Baiyou Company, Germany) outer joint edge fixation. In four cases, the Kirschner wire tension band was used to fix the fibular osteotomy and the cannulated nail to fix the upper tibiofibular joint (Baiyou Company, Germany).

### *Radiological Evaluation*

One patient underwent transfibular neck osteotomy and a 3‐mm step gradually appeared, but no significant abnormalities were found in the width of the platform and the lower limb force line. During the follow‐up period, no reduction loss was observed, and the width of the platform and the lower limb force line were normal. According to the Rasmussen radiological criteria, the scores at the last follow‐up were: for the combined anterolateral and posterolateral approach the score was 12–18 points, with an average of 16.00 ± 2.56 points; scores were excellent in 4 cases and good in 3 cases. Therefore, 100% of results were excellent or good. The transfibular neck osteotomy approach was 10–18 points, with an average of 15.25 ± 3.77 points, excellent in 2 cases, good in 1 case, and fair in 1 case. Therefore, 75% of results were excellent or good.

### *Knee Joint Function Evaluation*

There were no restrictions on daily activities in all cases after 12 months of follow‐up, and both the Lachman test and the Pivot‐shift test results were negative. All patients had complete knee extension. For the combined anterolateral and posterolateral approach, the knee flexion angle was 110° – 130°, with an average of 122.86° ± 7.56°. For the transfibular neck osteotomy approach, the knee flexion angle was 115°–130°, with an average of 120.00° ± 7.07°. For the combined anterolateral and posterolateral approach, the HSS knee score at the last follow‐up was 76–98 points, with an average of 88.43 ± 7.55 points; results were excellent in 5 cases and good in 2 cases Therefore, 100% of results were excellent or good. The transfibular neck osteotomy approach was 74–96 points, with an average of 87.25 ± 9.43 points; results were excellent in 3 cases and good in 1 case. Therefore, 100% of results were excellent or good.

### *Complications*

All the surgical incisions in this group were healed in one stage, and no complications such as wound infection, necrosis, and non‐union occurred. There was no internal fixation repulsion or looseness, and fracture of the upper tibiofibular fixation screw.

At the final follow‐up, no patients had developed symptoms of nonunion or malformation of the fibular osteotomy. There was no knee instability and dislocation of the upper tibiofibular joint.

One patient in whom the combined anterolateral and posterolateral approach was used showed numbness in the common peroneal nerve innervations and poor ankle joint and toe extension, and the symptoms gradually disappeared over 2 months after administration of nutritional peripheral nerve drugs. There were no symptoms of common peroneal nerve injury through the transfibular neck osteotomy approach.

For the transfibular neck osteotomy approach, 1 case gradually developed a 3‐mm step after operation, and pain occurred while walking. The posterolateral column fragmentation was extremely serious, and there were more bones in joint surface. Premature weight‐bearing causes articular surface re‐collapse. X‐ray showed that the width of the platform and the lower limb force line were acceptable. With symptomatic treatment using non‐steroidal anti‐inflammatory drugs and the reduction in the pain symptoms after weight‐bearing activities, there is no significant impact on daily life.

## Discussion

### *Characteristics of the Posterolateral Tibial Plateau Fracture*

Schatzker classification and three‐column classification are the most commonly used tibial plateau fracture classification systems. Lateral condyle of tibial plateau fractures involving the posterolateral column belong to Schatzker I, II, and III, or posterior or lateral column fractures in three‐column classification. However, these two classifications lack descriptions of fracture characteristics, and the clinical guidance is not specific enough. Chen *et al*.^23^ analyze 3D CT images. According to the characteristics of fracture injury, the classification of posterior condyle fractures of the tibial plateau has significance for the choice of surgical approach and internal fixation. It divides the posterior condyle fracture of the tibial plateau into five types, of which types II–V are involved in posterolateral fractures: type I, split fracture of posterior medial condyle; type II, split fracture of posterolateral condyle; type III, depression fracture of posterolateral condyle; type IV, split fracture of posterolateral condyle; and type V, split fractures of posteromedial condyle and depression fractures of posterolateral condyle. Further statistics showed that the incidence of type III (28.2%) and type V (35.9%) was higher in their classification^23^. Another study showed that^26^, ^27^ the posterolateral tibial plateau fractures were mostly belong to depression with/without split type; the posterior wall was comminuted and split fracture alone was rare. The 3D CT of this group showed that the height of the articular surface of the posterolateral platform was significantly reduced, and the bone of the joint surface was crushed. Therefore, in determining the treatment strategy for posterolateral column fractures, it is very important to reduce the collapsed articular surface. In this group in which transfibular neck osteotomy was used, the degree of crushing and collapse of the articular surface of the posterolateral plateau was significantly higher than that for the combined anterolateral and posterolateral approach. The deepest collapse was 20.3 mm, and the split fracture was more serious. Gao *et al*.^28^ measured the axial angle and sagittal angle of the fracture line of the posterolateral fracture block, suggesting that the fracture line of the fracture block is biased to the coronal position and is prone to displacement under vertical shear stress. Therefore, it is necessary to place the screw in the vertical fracture line of the posterior support plate, and the posterior support plate fixation fulfills biomechanical strength requirement. Analysis and understanding of these fracture characteristics can provide a better understanding of the pathology of the fracture, which helps in selecting the appropriate surgical approach and internal fixation.

### *The Choice of Two Surgical Approaches and the Internal Fixation*

The results of this study showed that both the combined anterolateral and posterolateral approach and the transfibular neck osteotomy approach have a satisfactory surgical outcome in the treatment of lateral condyle of tibial plateau fractures involving the posterolateral column. There were no significant differences in operation time, surgical blood loss, fracture healing time, postoperative imaging scores, and knee function evaluation between the two groups. However, for fracture exposure, the transfibular neck osteotomy approach is more visible and clear. We pulled the fibular head to the proximal end to completely remove the blocking effect of the fibular head on the posterolateral platform. The lateral tibial plateau is restored under the direct vision of the anterolateral and posterolateral, and the internal fixation can also be varied. Solomon *et al*. 21 considered this approach to be suitable for the lateral tibial plateau split depression fracture of lateral tibial plateau with comminuted fracture of posterolateral tibial plateau. In this group, the patients with transfibular neck osteotomy had the characteristics of split depression fracture, and the articular surface was crushed with obvious collapse and bone block separation and displacement; the posterior wall splitting fracture line was large and involved the upper tibiofibular syndesmosis area. Studies have shown that the distance from the surface margin of the fibular capitulum joint to the lateral edge of the tibial plateau joint is approximately 12 mm^29^, ^30^. For collapses beyond this range, it is very difficult to reduce the posterior column after being squeezed by the posterior femoral condyle the knee flexed. The degree of posterolateral articular surface collapse of this group of osteotomy cases exceeded this standard, and the deepest was 20.3 mm. In addition, the resetting of the posterior wall split fracture involving the upper tibiofibular syndesmosis area was affected by the complete occlusion of the fibular head. Therefore, we took the articular surface collapse >12 mm and the posterior wall splitting fracture line involving the upper tibiofibular syndesmosis area as the main indication for the fibular osteotomy approach.

We summarize the surgical indications of the transfibular osteotomy approach as follows: (i) depression fracture and split fracture both exist simultaneously exist and need to be treated; (ii) the posterolateral articular surface damage is accompanied by bone separation and displacement, and the collapse depth is >12 mm, which requires a larger range and clear exposure; (iii) the posterolateral fracture range involves a large range of the posterolateral column, especially the upper tibiofibular syndesmosis area of the line connecting the anterior and posterior margin of the fibular head to the midpoint of the plateau; and (iv) after the reduction of the posterolateral collapsed articular surface bone, the bones need to be fixed with a more precise articular surface support such as a “ring plate.”

Although the posterolateral approach can directly reveal the posterolateral column, we found that this approach has very limited exposure to the posterolateral platform. Only the edge area of the posterolateral platform and posterior walls can be observed. It is not clear whether the articular surface is anatomically reduced or not, and with the narrow operating space, it is also difficult to reset the comminuted articular bone. The anterolateral approach is relatively simple and safe, and the entire position of the lateral tibial plateau fracture can be fully revealed and restored with appropriate posture and reasonable retraction during surgery 29, 31. We realized that the reduction of the fracture and the fixation of the lateral plate were mainly performed by the anterolateral approach, while in the majority of cases, the posterolateral approach was only used for simple placement of the posterior plate to achieve support fixation. Compared with the transfibular osteotomy approach, the combined anterolateral and posterolateral approach is more suitable for the following conditions: (i) the anterolateral column and the posterolateral column are involved in the fracture, and the posterior support plate is required for fixation; (ii) the posterolateral platform surface collapse <12 mm, the degree of comminution is lighter, the fracture block separation is not obvious, and the anterolateral approach can achieve satisfactory fracture reduction; (iii) the split fracture did not involve the upper tibiofibular syndesmosis area; and (iv) the anterolateral column fracture involved the metaphysis, requiring longer plate fixation^32^.

In general, anterolateral “L” shaped and posterolateral “T” shaped double plate fixation can provide confirm the biomechanical requirement for the lateral column and posterolateral column fractures^17^, ^18^. The range of expose of this approach is more large, the anterior lateral “L”‐shaped steel plate can be more posterior, thereby increasing the fixed range of the posterior margin of the posterolateral column. Fracture reduction alone is not sufficient when the articular surface is severely crushed and collapsed with multiple cleft fracture lines. In this group, 1 case gradually developed a 3‐mm step after operation. The posterolateral column fragmentation of the patient was extremely serious, and there were more bones in the joint surface. The premature weight‐bearing caused the articular surface to collapse again, indicating the necessity of direct plate fixation on the posterior side. The use of the periarticular edge plate can also make up for the above shortcomings. The edge plate technology can not only enable screw placement to the posterolateral side of the platform but also provide support for the debris, maintaining the comminuted cortex and the joint surface edge. We used a 1/4 tube‐shaped plate as the periarticular plate. This plate is small and easy to shape. It is suitable for the bony space above the fibular head and the bone‐ligament space, and does not affect the upper tibiofibular joint. After the articular surface bone was restored, the edge plate surrounded the bone block; a longer diameter 2.7‐mm screw was used, which was closer to the cartilage surface to fix the collapsed bone block. We made it an integral whole and then combined the lateral “L” shape and the posterolateral “T” shape double plate fixation. In this group, two cases were treated with marginal plates and achieved good clinical results. There was no loss of reduction after surgery. For large‐area fractures of the posterior articular surface of the tibial plateau, edge impact, posterior cortical wall comminution, and subchondral epiphyseal depression may be better treated with “ring plates”^33^.

The upper tibiofibular joint is amphiarthrosis. After the fibula reduction in the case of the transfibular osteotomy, we fixed the upper tibiofibular joint by inserting a screw in the posterolateral platform through the fibular head. This can increase the stability of the upper tibiofibular joint and facilitate the repair of the upper tibiofibular ligament. It also strengthens the fixation strength of the posterolateral plateau with the support of the fibular^34^.

### *Surgical Complications and Prevention*

In theory, the transfibular osteotomy approach has complications, such as common peroneal nerve injury, nonunion of the fibular, affecting the’ the stability of the upper tibiofibular joint. Solomon^21^ believes that the complications associated with the transfibular osteotomy approach include only iatrogenic common peroneal nerve injury and nonunion of fibular osteotomy. However, the current literature indicates that the above complications do not occur with transfibular osteotomy^11^, ^20^, ^21^. The last follow‐up result for this group of patients was negative for both the Lachman test and the Pivot‐shift test. No patients developed symptoms of knee instability. In the final imaging examination of Pires *et al*.,^11^ all fractures healed, and no patients developed symptoms of non‐union or malunion of the transfibular osteotomy. Although the time of healing of the fibular osteotomy was not specifically recorded in this group of patients, it was confirmed that delayed healing and non‐union did not occur. In this group, there was no common peroneal nerve injury after the transfibular osteotomy approach. We believe that the following points should be noted in controlling complications: (i) thoroughly dissect the common peroneal nerves and strengthen the protection; (ii) carefully suture the anterior and posterior tibiofibular ligaments and joint capsules; (iii) upper tibiofibular joint fixation can increase the stability of the upper tibiofibular joint; (iv) to reduce the stimulation to common peroneal nerve by the end of bone after osteotomy; and (v) the use of >80mm extra‐long screws to fix the fibular osteotomy can increase the fixation stability and facilitate fracture healing^11^.

In summary, a lateral tibial plateau fracture involving the posterolateral column has clinical features of a severely complex tibial plateau fracture. Orthopaedic surgeons have explored multiple surgical approaches, reductions, and fixations for this type of fracture. The combined anterolateral and posterolateral approach can repair the fracture of the lateral column and the posterior lateral column, and complete the fixation of the lateral and posterior double plates. The main advantage of the transfibular osteotomy approach is that the anatomical reduction of the joint can be performed intuitively, and it is more suitable for the treatment of posterolateral column fractures of some special types of tibial plateau. The periarticular margin plate and the upper tibiofibular joint fixation provide a new internal fixation technique. There are some limitations of this study, including few sample cases, no control group, follow‐up time was short, this was a retrospective case analysis, it may influence the conclusion. More forward‐looking studies on injury mechanisms and fracture morphology, and involving biomechanical assessments will help determine the surgical techniques used and the true complication rates, and verify their safety and utility.
